# Dr. Franklin E. Burr

**Published:** 1898-10

**Authors:** 


Dr. Franklin E. Burr, U. of B. '90, died at his home at Greely, Col., September 6, 1898, aged 34 years. Dr. Burr was born at Lindley, N. Y., January 5, 1864, and at the age of 16 was left by the death of his father to his own resources. Afterward he went to Cleveland, O., where he engaged in the lumber business, devoting evenings and holidays to the pursuit of education. After three years spent in this way he entered Buffalo University Medical College, from which he graduated in 1890.

Dr. Burr located first in Connecticut, where he remained a year; then at Corning, N. Y., where he practised for two years and a half. His health then failed and he went to New Mexico and Wyoming, finally locating at Greely, Col., where he remained until his death. He soon acquired a reputation for medical and surgical skill, obtaining the confidence of the community, and died regretted by a large clientéle.

